# Incorporation of selenium nanoparticles into mineral trioxide aggregate used as a direct pulp capping material

**DOI:** 10.2340/biid.v13.45749

**Published:** 2026-04-10

**Authors:** Njwan Fadhel Shehab, Hana Khaleel Ismail, Nadia H. Hasan

**Affiliations:** a Department of Conservative Dentistry, College of Dentistry, University of Mosul, Mosul, Iraq; b Department of Pathology and Poultry Disease, College of Veterinary Medicine, University of Mosul, Mosul, Iraq

**Keywords:** Selenium nanoparticles, inflammation, reparative dentin, necrosis, direct pulp capping

## Abstract

**Introduction:**

This study evaluated the histopathological response of the dental pulp following direct pulp capping (DPC) using Mineral Trioxide Aggregate Repair High Plasticity (MTA Repair HP) supplemented with green-synthesized selenium nanoparticles (SeNPs). The objective was to determine how SeNP concentration influences the biological behavior of MTA HP and to identify the dose that best supports pulp healing compared with MTA HP alone.

**Methods:**

Twelve male dogs with 132 teeth were included. Twelve teeth served as negative controls, while 120 teeth were assigned to five groups: MTA HP alone or MTA HP combined with 0.5%, 1%, 1.5%, or 2% (w/w) SeNPs (*n* = 24 per group). Standardized pulp exposures were created and treated according to group allocation. Samples were collected at 7, 14, 30, and 60 days (*n* = 6 per interval) for histopathological assessment of inflammation, necrosis, and reparative dentine formation.

**Results:**

The group treated with 1% SeNP had the best results. They displayed very little inflammation, no signs of tissue necrosis, and started forming reparative dentine as early as day 14. While inflammation diminished over time in all the groups, the statistical analysis showed that the different SeNP levels made a difference in how much inflammation occurred, how much tissue necrosis there was, and the quality of the new dentine.

**Conclusion:**

Adding SeNP to MTA HP depended on the amount used. The 1% version presented the best biocompatibility and assisted pulp tissue heal better. On the other hand, higher amounts triggered more inflammation. Overall, these results recommend that 1% SeNP-enhanced MTA HP could be a choice for direct pulp capping, offering better healing and clinical results.

## Introduction

Direct pulp capping (DPC) is the preferred treatment of an exposed dental pulp, aiming to maintain pulp vitality and promote dentinogenesis by inducing undifferentiated mesenchymal cells to form reparative dentine. This approach is indicated only in vital teeth without spontaneous pain, mobility, or signs of necrosis, and contributes to increased tooth longevity and prevention of tooth loss [1].

Mineral Trioxide Aggregate (MTA) is widely used for DPC; however, histological studies have reported that MTA can increase pulp inflammatory cell populations, enlarge necrotic areas due to its high alkalinity, and stimulate interleukin release [2]. Oxidative stress, caused by excess free radicals, damages nucleic acids and proteins, disturbing biological processes. Both endogenous and exogenous antioxidants can limit this damage by preventing reactive oxygen species (ROS) formation [3]. The incorporation of therapeutic nano-additives into MTA has been proposed to enhance odontoblastic differentiation from dental pulp stem cells (DPSCs) by downregulating ROS [4].

Selenium is an essential trace element involved in enzymes and proteins that protect cells from damage and infection. In nanoparticle form, selenium exhibits greater antioxidant activity, biocompatibility, and anti-inflammatory properties than its conventional form, owing to its increased surface-to-volume ratio [5]. Selenium nanoparticles (SeNPs) reduce inflammation by inhibiting prostaglandin E2, which activates pro-inflammatory cytokines, and modulate ROS levels while enhancing cell adhesion and differentiation. Their biocompatible antioxidant properties have supported their use in medical applications for hard tissue regeneration [6].

Reducing the activity of proinflammatory cytokines is an important strategy in controlling inflammation, as their excessive chronic production contributes to inflammatory diseases [7]. Green nanotechnology, which uses plant extracts and microorganisms, has gained attention for its biocompatibility [8]. Histopathological evaluation of inflammatory responses to MTA HP remains limited [9], leading to the hypothesis that green-synthesized SeNPs incorporated into MTA may improve DPC outcomes. This in vivo study evaluated the pulpal response in dogs to MTA HP modified with varying concentrations of SeNPs (0.5%, 1%, 1.5%, and 2% w/w) after DPC. Histopathological assessments were conducted at 7, 14, 30, and 60 days post-treatment and compared to a control group treated with MTA HP alone, aiming to assess the potential of SeNPs-enhanced MTA for pulpal healing and regeneration.

## Materials and methods

### Ethical committee approval

On January 29, 2023, the Research Ethics Committee of the College of Dentistry, at the University of Mosul, Iraq, approved the use of dogs in the study, granting protocol authorization with reference number UoM. Dent/H.DM.15/23.

### In vivo animal study

The green-synthesized SeNPs powder was obtained from Nano Research Elements Company (India) upon request (99.9% purity, an average particle size of approximately 50 nm, and a predominantly spherical morphology). This animal study evaluated the effects of MTA HP modified with SeNPs on canine pulp tissue, comparing it with commercially available MTA HP. Procedures followed ISO 7405:2008 standards and methodologies from previous pulp-capping studies [10, 11].

### Protective measurement

Before animal contact, the researcher received preventive rabies vaccination per WHO guidelines (WHO, 2018), administered intramuscularly in two doses on days 0 and 7. Protective measures included a lab coat, gown, double gloves, double masks, eye protection, and a face shield. All instruments were sterilized in an autoclave at 121°C and 15 psi for 15 min.

### Animal collection, selection, and housing

Eighteen healthy male dogs of local breeds, weighing between 15 and 20 kilograms, aged around 12 to 18 months, with 198 teeth. However, six of them had to be excluded because of loss or problems during processing, so in the end, only twelve dogs were used for the study. The dogs were kept individually in ventilated cages, checked by a vet, and given vaccinations with Biocan DHPPi + LR to protect them against various viruses and rabies. The dogs were kept at the College of Veterinary Medicine in clean, healthy conditions. They were fed a diet of chicken and rice twice a day, along with tap water, and were regularly checked by veterinarians. After about a month, the dental work was done at the College of Dentistry, and the surgical procedures took place at Veterinary Medicine. Only dogs that had healthy gums and teeth, confirmed through X-rays, were included in the study.

### General anesthesia and dog preparation

Before the dental procedure, dogs were fasted for 12 h without food and 6 h without water and weighed to calculate anesthetic dosage. Premedication involved intramuscular atropine sulfate (0.04 mg/kg) 15 min before induction to reduce secretions, maintain heart rate, and prevent arrhythmia [12]. General anesthesia was induced with a combined intramuscular injection of ketamine hydrochloride 10% (10 mg/kg) and xylazine 2% (3 mg/kg), achieving anesthesia within 5–10 min, with maintenance doses administered every 30 min [13].

### Animal teeth selections and sample grouping

**Figure 1:**
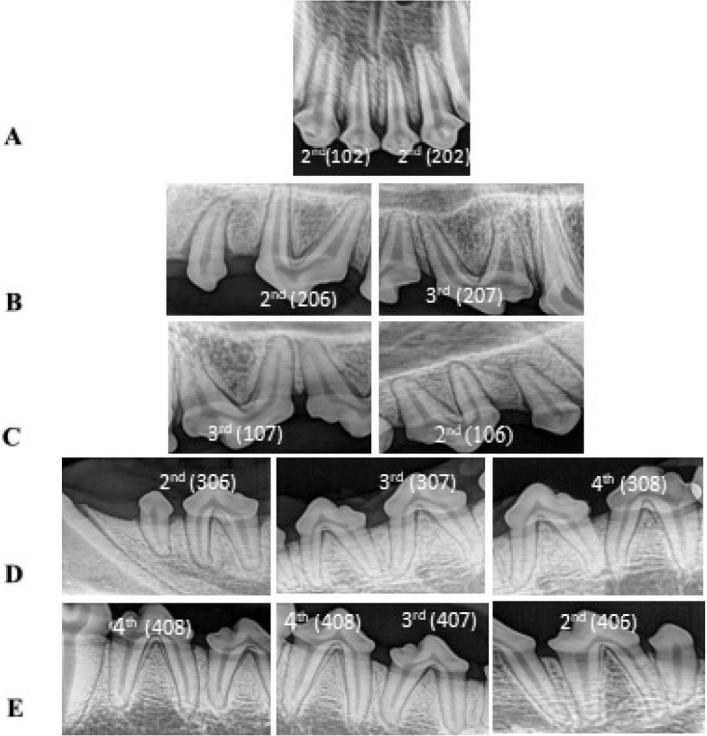
Preoperative periapical radiographs for the selected teeth before treatment. (**a**) Upper right or left 2nd incisors; (**b**) upper left 2^nd^ and 3^rd^ premolars; (**c**) upper right 2^nd^ and 3^rd^ premolars; (**d**) lower left 2^nd^, 3^rd^ and 4^th^ premolars; (**e**) lower right 2^nd^, 3^rd^ and 4^th^ premolars.

A total of 132 teeth were used in this study, with 11 teeth selected from each dog, including maxillary and mandibular premolars and the maxillary second incisor. Teeth were required to have intact, healthy crowns without fractures or caries. Pre-operative digital radiographs (60 kV, 2 mA, 1.0 sec) were taken to ensure fully developed root apices, absence of calcification or root resorption, and healthy apical tissues, as shown in Figure 1.

The teeth were divided into a positive control group, with pulps exposed and capped using MTA HP, and experimental groups capped with MTA HP containing SeNPs at 0.5%, 1%, 1.5%, and 2% w/w. Each group included 24 teeth, with 6 teeth per evaluation time point (7, 14, 30, and 60 days), using two teeth per dog. Twelve healthy, unexposed teeth served as negative controls to assess normal pulp histology. The study employed a split-mouth design, evaluating all groups within the same animals on opposite sides of the mouth [10, 14].

The required sample size was determined before the study based on previous animal research on pulp-capping materials using a split-mouth design and histological evaluation. Using G*Power software, a medium effect size (*f* = 0.3), a significance level of 0.05, and 72% statistical power were applied. This calculation indicated that six teeth per group for each time point would be sufficient, resulting in a total of 24 teeth per group across the four evaluation periods and aligning with earlier studies in the field [10, 11].

### Operative procedure and specimen collections

The DPC procedure was performed as a single-visit vital pulp therapy under general anesthesia with the dog positioned supine and the teeth isolated using a rubber dam. Teeth and surrounding tissues were disinfected with 0.2% chlorhexidine and 10% povidone-iodine. Class V cavities were prepared on the labial/buccal surfaces using a high-speed turbine with water cooling, and pulp exposure was created mechanically with a sharp explorer under 3.5× magnification, controlling exposure size to approximately 0.5 mm [10, 14]. Hemorrhage confirmed exposure, followed by saline rinsing and bleeding control with sterile cotton pellets to preserve pulp vitality. MTA HP was mixed per manufacturer instructions, applied to the exposure site, and sealed with chemically cured glass ionomer material. Following the pulp capping procedure, the animals were monitored daily during the healing period. At the end of the experimental period, the animals were deeply anesthetized and humanely euthanized. The maxilla and mandible were then carefully separated, followed by soft tissue removal. Surgical incisions reflected buccal and lingual soft tissues, and jaws were dissected behind the molars using a 0.5 mm disk with saline coolant. Each jaw was halved at the midline, fixed in a bench vice, and experimental teeth with surrounding bone were segmented using a 0.2 mm disk under saline irrigation. Specimens were preserved in 10% neutral-buffered formalin for histopathological evaluation.

### Histological process

Tissue samples were fixed in 10% neutral-buffered formalin for 14 days, decalcified in 10% ethylene diamine tetraacetic acid (EDTA) at 37°C for 6 months with weekly X-ray monitoring. After rinsing, specimens were dehydrated through graded ethanol, cleared with xylene, and embedded in paraffin wax. Sections of 5 μm were cut using a rotary microtome through the pulp exposure site. Slides were stained with Harris’s hematoxylin and eosin, dehydrated, cleared, mounted, and prepared for microscopic examination [10, 12].

### Examination and digital histomorphometric analysis

All histological sections were coded, and the histopathologist evaluating the slides was blinded to the experimental group assignments, ensuring unbiased assessment of inflammatory response, necrosis, and dentine bridge formation. Histopathological evaluation of stained pulp tissue slides was performed using digital light microscopy at ×10, and ×40 magnifications. Six readings per group and time point were analyzed with Optika software for inflammatory response, necrosis, and reparative dentine formation, following criteria by Hoseinifar et al. [15], and Octiara et al. [16]. Inflammatory cell infiltration was quantified using a nine-square grid method.

The inflammatory response intensity was graded according to Octiara et al. [16].

- **Score 1:** no or slight inflammatory response.
- **Score 2:** mild (mean number of inflammatory cells < 10).
- **Score 3:** moderate (mean number of inflammatory cells 10–25).
- **Score 4:** severe (mean number of inflammatory cells > 25).

The assessment of necrosis was graded according to Hoseinifar et al. [15].

- **Score 0:** for absence of necrosis.
- **Score 1:** for presence of necrosis.

### Statistical analysis

Data were analyzed using SPSS v22. Normality was assessed with Shapiro-Wilk’s test, revealing non-normal distribution; thus, nonparametric tests were applied. Kruskal-Wallis and Dunn tests compared groups at each time point, while Friedman’s test assessed changes over time within groups. Significance was set at *P* ≤ 0.05, with *P* < 0.01 considered highly significant.

## Results

### Macroscopic evaluation (clinical examination) and radiographic examination

**Figure 2:**
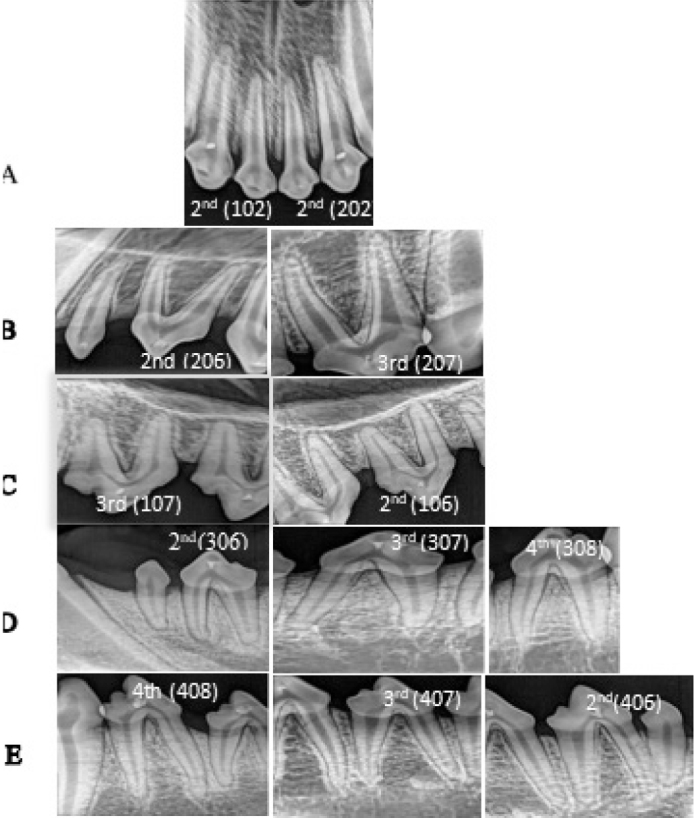
Postoperative periapical radiographs for pulp capped exposed teeth. (**a**) Upper right or left incisors; (**b**) upper left 2nd and 3rd premolars; (**c**) upper right 2nd and 3rd premolars; (**d**) lower left 2nd, 3rd and 4th premolars; (**e**) lower right 2nd, 3rd and 4th premolars after treatment.

Throughout the study, clinical examinations showed no behavioral changes or signs of dental pathology in treated dogs, with normal gingival appearance. Radiographs revealed no periapical lesions or root resorption at any time point (Figure 2).

### Microscopic examination

#### Histopathological comparisons

The negative control group with normal pulp tissue exhibited typical histological features, including an intact odontoblast layer, well-defined cell-free and cell-rich zones, and normal vascularization.

#### Seven days following direct pulp capping

**Figure 3:**
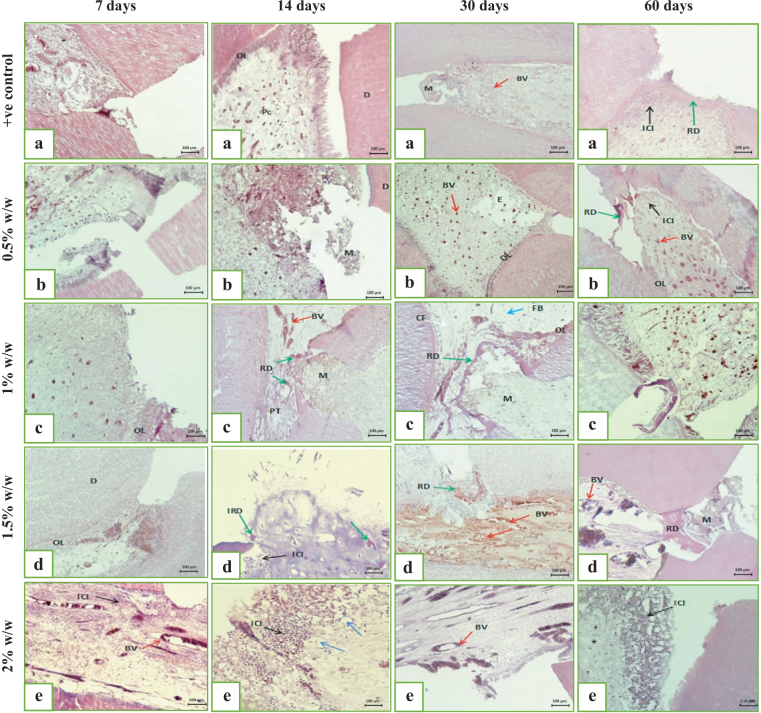
A digital micrograph representing the intensity of the inflammatory response from the pulp capped with (a) MTA HP/+ve control group, (b) MTA HP incorporated 0.5% w/w SeNPs, (c) MTA HP incorporated 1% w/w SeNPs, (d) MTA HP incorporated 1.5% w/w SeNPs, and (e) MTA HP incorporated 2% w/w SeNPs for a 7, 14, 30 and 60 days observation period, showing inflammatory cell infiltration (ICI) (black arrow), blood vessels (BV) (red arrow), collagen fiber (CF) (white arrow), and fibroblast (FB) (blue arrow), reparative dentine (RD) (green arrow), initial reparative dentine (IRD) (green arrows), and thickened blood vessel wall (TBV) (red arrow) (H&E, ×10). dentine (D), central pulp core (Pc), and odontoblastic layer (OL).

**Figure 4:**
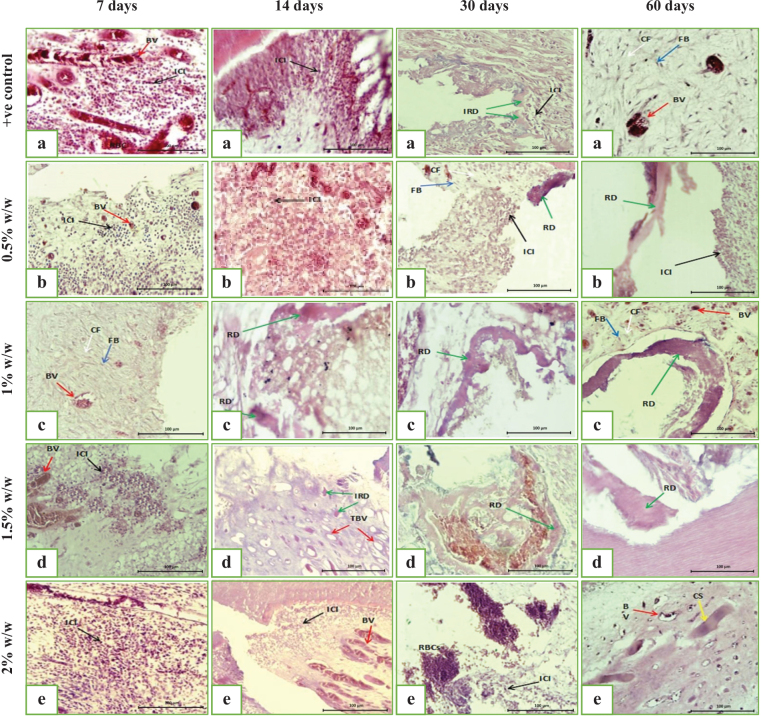
A digital micrograph representing the intensity of the inflammatory response from the pulp capped with (a) MTA HP/+ve control group, (b) MTA HP incorporated 0.5% w/w SeNPs, (c) MTA HP incorporated 1% w/w SeNPs, (d) MTA HP incorporated 1.5% w/w SeNPs, and (e) MTA HP incorporated 2% w/w SeNPs for a 7, 14, 30 and 60 days observation period, showing inflammatory cell infiltration (ICI) (black arrow), blood vessels (BV) (red arrow), collagen fiber (CF) (white arrow), and fibroblast (FB) (blue arrow), reparative dentine (RD) (green arrow), initial reparative dentine (IRD) (green arrows), and thickened blood vessel wall (TBV) (red arrow) (H&E, ×40). dentine (D), central pulp core (Pc), and odontoblastic layer (OL).

At 7 days post-DPC, all groups showed disruption of the odontoblastic layer near the exposure site and loss of normal pulp architecture. The positive control group (MTA HP) demonstrated severe inflammatory cell infiltration, necrosis near the exposure, dilated congested vessels, extravasated RBCs, and absence of reparative dentine formation (Figures 3a and 4a).

In the experimental groups with MTA HP containing SeNPs, varying inflammatory responses were observed:

- 0.5% SeNPs: Moderate inflammation with localized necrosis and dilated vessels; no dentine bridge (Figures 3b and 4b).
- 1% SeNPs: Highly vascularized pulp with minimal inflammation, preserved connective tissue, but no reparative dentine (Figures 3c and 4c).
- 1.5% SeNPs: Moderate inflammation, edema, necrotic foci, vascular congestion, and extravasated RBCs without dentine bridge formation (Figures 3d and 4d).
- 2% SeNPs: Severe inflammatory infiltration, tissue necrosis, and vascular congestion; no reparative dentine observed (Figures 3e and 4e).

#### Fourteen days following direct pulp capping

At 14 days, the positive control group (MTA HP) showed moderate inflammatory infiltration with superficial necrosis and increased vascularization; inflammation was reduced compared to day 7, but no reparative dentine formation was observed (Figures 3a and 4a).

The experimental group with 0.5% SeNPs exhibited localized moderate inflammation and necrotic foci beneath the exposure site, with decreased inflammation compared to day 7 and well-organized deeper pulp tissue containing fibroblasts and collagen (Figures 3b and 4b).

The 1% SeNPs group demonstrated partial reparative dentine bridge formation continuous with the exposure edges, absence of inflammatory cells, restored odontoblastic layer except at the exposure site, and vascular congestion (Figures 4c and 5c).

The 1.5% SeNPs group showed initial reparative dentine deposition, mild inflammation, vascular wall thickening, and localized odontoblastic disruption at the exposure site (Figures 4d and 5d).

The 2% SeNPs group displayed disrupted pulp architecture with moderate inflammation, vascular congestion, vacuolation, and tissue necrosis (Figures 4e and 5e).

#### Thirty days direct pulp capping

At 30 days, the positive control group (MTA HP) exhibited mild localized inflammation, initial hard tissue deposition, and vascular congestion (Figures 4a and 5a). The 0.5% SeNPs group showed mild inflammation at the exposure site, vascular congestion, edema, well-organized deeper pulp with fibroblasts, and early reparative dentine formation (Figures 4b and 5b). While 1% SeNPs group demonstrated minimal inflammation, and complete calcified dentine bridge formation at the exposure site (Figures 4c and 5c).

The 1.5% SeNPs group presented slight inflammation, dilated congested vessels, complete dentine bridge formation, along with pulp tissue degeneration and hemorrhage (Figures 4d and 5d).

The 2% SeNPs group revealed moderate inflammation, disrupted pulp architecture, vascular congestion with RBC extravasation, and absence of reparative dentine (Figures 4e and 5e).

#### Sixty days following direct pulp capping

The positive control group (MTA HP) showed mild localized inflammation, a complete reparative dentine bridge closing the exposure site, partial restoration of the odontoblastic layer, and organized deeper pulp tissue with vascular congestion (Figures 4a and 5a).

The 0.5% SeNPs group exhibited mild inflammation, dilated congested vessels, and continuous reparative dentine bridge formation (Figures 4b and 5b).

The 1% SeNPs group demonstrated no inflammation, a uniform and complete reparative dentine bridge, restored odontoblastic layer, increased vascularization, and advanced pulp healing (Figures 4c and 5c).

The 1.5% SeNPs group showed minimal inflammation, complete reparative dentine bridge with variable calcification, hemorrhagic areas, vascular congestion, and disruption of the odontoblastic layer (Figures 4d and 5d).

The 2% SeNPs group presented moderate inflammation, necrosis, absence of reparative dentine bridge, and multiple calcified deposits suggestive of pulp tissue metaplasia (Figures 4e and 5e).

### Statistical comparisons

#### Inflammatory response

1. Comparison of different groups within the same time interval

Statistical analysis using Kruskal-Wallis and Dunn’s post hoc tests (*P* ≤ 0.05) revealed significant differences in inflammatory responses and tissue necrosis among groups at all observation periods (Tables 1 and 2).

**Table 1:** Analysis of inflammatory intensity scores (Kruskal-Wallis and Friedman’s tests).

Time/days	Scores	Groups					*P*-value (Between groups)
		Gp I *N* = 24 *n* = 6	Gp II *N* = 24 *n* = 6	Gp III *N* = 24 *n* = 6	Gp IV *N* = 24 *n* = 6	Gp V *N* = 24 *n* = 6	

		Percentage of inflammatory cell scores (%)					
7 days (T1)	1	-	-	50.0%	-	-	0.000*
	2	-	-	50.0%	16.7%	-	
	3	83.3%	100%	-	83.3%	-	
	4	16.7%	-	-	-	100%	
		**B, b**	**B, b**	**A**	**B, b**	**C, b**	
14 days (T2)	1	-	-	83.3%	-	-	0.000*
	2	-	33.3%	16.7%	50.0%	-	
	3	100%	66.7%	-	50.0%	100%	
	4	-	-	-	-	-	
		**B, b**	**B, ab**	**A**	**B, b**	**B, a**	
30 days (T3)	1	-	-	100%	66.7%	-	0.000*
	2	50.0%	50.0%	-	33.3%	-	
	3	50.0%	50.0%	-	-	100%	
	4	-	-	-	-	-	
		**B, ab**	**B, ab**	**A**	**A, a**	**B, a**	
60 days (T4)	1	-	-	100%	66.7%	-	0.000*
	2	100%	100%	-	33.3%	33.3%	
	3	-	-	-	-	66.7%	
	4	-	-	-	-	-	
		**B, a**	**B, a**	**A**	**A, a**	**B, a**	
***P*-value (Within group)**		0.003*	0.008*	0.066 NS	0.002*	0.001*	

*N*: total number of samples per group across all time points, *n*: number of samples per group at each time point. Differences among groups at the same time point were analyzed using the Kruskal–Wallis test. Different capital letters indicate statistically significant differences among groups at the same time point. Differences across time points within each group were analyzed using Friedman’s test. Different lowercase letters indicate statistically significant differences over time within the same group. **Desc:** Score 1= no to slight inflammatory cells; Score 2= mild; Score 3= moderate; Score 4= severe. **Groups: I** = control positive, **II** = 0.5%w/w, **III** = 1%w/w, **IV** = 1.5%w/w, **V** = 2%w/w. NS: non-significant.

* *P* ≤ 0.05 means significant variation exists.

**Table 2:** Analysis of tissue necrosis scores (Kruskal-Wallis and Friedman’s tests).

Time/days	Scores	Groups					*P*-value (Between groups)
		Gp I *N* = 24 *n* = 6	GpII *N* = 24 *n* = 6	Gp III *N* = 24 *n* = 6	Gp IV *N* = 24 *n* = 6	Gp V *N* = 24 *n* = 6	

		Percentage of tissue necrosis scores (%)					
7 days (T1)	0	0	0	100%	16.7%	0	0.000*
	1	100%	100%	0	83.3%	100%	
		**B, b**	**B, b**	**A**	**B, b**	**B**	
14 days (T2)	0	0	33.3%	100%	50%	0	0.002*
	1	100%	66.7%	0	50%	100%	
		**B, b**	**B, ab**	**A**	**AB, ab**	**B**	
30 days (T3)	0	50%	50%	100%	100%	0	0.002*
	1	50%	50%	0	0	100%	
		**AB, ab**	**AB, ab**	**A**	**A, a**	**B**	
60 days (T4)	0	100%	100%	100%	100%	33.3%	0.001*
	1	0	0	0	0	66.7%	
		**A, a**	**A, a**	**A**	**A, a**	**B**	
***P*-value (Within group)**		0.003*	0.008*	1.000 NS	0.007*	0.112 NS	

*N*: total number of samples per group across all time points, *n*: number of samples per group at each time points Differences among groups within the same time points were analyzed using the Kruskal–Wallis test. Different capital letters indicate statistically significant differences among groups within the same time points that are represented by Dunn’s post hoc tests. Differences across time periods within each group were analyzed using Friedman’s test. Different lowercase letters indicate statistically significant differences among time periods within the same group. **Groups:** I = control positive, II = 0.5%w/w, III = 1%w/w, IV = 1.5%w/w, V = 2%w/w. **Tissue necrosis scores**: 0 = absence of necrosis, 1 = denaturation of proteins. NS: non-significant.

* *P* ≤ 0.05 means significant variation exists.

- **At 7 days**, control positive, 0.5%w/w, and 1.5%w/w showed moderate to mild inflammation with no significant differences among them; 1%w/w exhibited significantly less inflammation and necrosis, while 2%w/w had severe inflammation distinct from others.
- **At 14 days**, inflammation decreased with 0.5%w/w, 1.5%w/w, 2%w/w and control group showing no significant differences; 1%w/w maintained significantly lower inflammation and necrosis.
- **At 30 days**, inflammation further declined, with groups control, 0.5%w/w, and 2%w/w showing moderate responses; groups 1%w/w and 1.5%w/w exhibited absent to mild inflammation, significantly differing from others.
- **At 60 days**, mild inflammation persisted in some groups; control group and 0.5%w/w showed no significant difference, while group 2%w/w had higher tissue reaction. Groups 1%w/w and 1.5%w/w displayed minimal inflammation and were significantly different from others. Tissue necrosis persisted mainly in group 2%w/w (66.7%), with no necrosis observed in group 1%w/w throughout the study.

2. Comparison between different time points within the same group

Using Friedman’s test with pairwise comparisons (*P* ≤ 0.05), significant temporal changes in inflammatory response and tissue necrosis were observed in most groups (Tables 1 and 2).

- **Group I (control MTA HP):** Moderate inflammation at 7 days improved progressively to mild at 60 days, with significant differences over time (*P* = 0.003). Tissue necrosis decreased significantly, becoming absent by 60 days (*P* = 0.003).
- **Group II (0.5% w/w SeNPs):** Similar pattern to Group I with inflammation reducing from moderate to mild over 60 days (*P* = 0.008). Tissue necrosis also declined significantly, being absent by 60 days (*P* = 0.008).
- **Group III (1% w/w SeNPs):** No significant changes in inflammation over time (*P* = 0.066), and no tissue necrosis observed at any interval (*P* = 1.000).
- **Group IV (1.5% w/w SeNPs):** Significant reduction in inflammation and necrosis over time (*P* = 0.002 and *P* = 0.007, respectively), with necrosis absent after 30 days.
- **Group V (2% w/w SeNPs):** Severe inflammation at 7 days that decreased to moderate-mild by 60 days (*P* = 0.001); tissue necrosis showed no significant change during the period (*P* = 0.112).

#### Dentine bridge formation

1. Comparison of different pulp capping groups on reparative barrier score at the same time interval

The Kruskal-Wallis test followed by Dunn’s post hoc analysis (*P* ≤ 0.05) was used to assess differences in reparative dentine bridge formation among groups at each time point (Table 3).

**Table 3:** Analysis of reparative dentine bridge scores (Kruskal-Wallis and Friedman’s tests).

Time/days	Scores	Groups					*P*-value (Between groups)
		Gp I *N* = 24 *n* = 6	GpII *N* = 24 *n* = 6	Gp III *N* = 24 *n* = 6	Gp IV *N* = 24 *n* = 6	Gp V *N* = 24 *n* = 6	

		Percentage of score for hard tissue formation (%)					
7 days (T1)	1	-	-	-	-	-	1.000 NS
	2	-	-	-	-	-	
	3	-	-	-	-	-	
	4	100%	100%	100%	100%	100%	
		**b**	**b**	**c**	**b**		
14 days (T2)	1	-	-	-	-	-	0.000*
	2	-	-	66.7 %	-	-	
	3	-	-	33.3 %	50 %	-	
	4	100%	100%	-	50 %	100%	
		**B, b**	**B, b**	**A, b**	**B, b**	**B**	
30 days (T3)	1	-	-	100%	83.3 %	-	0.000*
	2	-	-	-	16.7 %	-	
	3	50 %	50%	-	-	-	
	4	50 %	50%	-	-	100%	
		**B, b**	**B, b**	**A, a**	**A, a**	**B**	
60 days (T4)	1	50 %	66.7%	100%	83.3 %	-	0.000*
	2	50 %	33.3%	-	16.7 %	-	
	3	-	-	-	-	16.7 %	
	4	-	-	-	-	83.3 %	
		**A, a**	**A, a**	**A, a**	**A, a**	**B**	
***P*-value (Within group)**		0.001*	0.001*	0.000*	0.001*	0.392 NS	

*N*: total number of samples per group across all time points, *n*: number of samples per group at each time point. Differences among groups at each time point were analyzed using the Kruskal–Wallis test. Within-group differences across time points were analyzed using Friedman’s test. Different uppercase letters indicate statistically significant differences among groups at the same time point. Different lowercase letters indicate statistically significant differences among time points within the same group. **Groups:** I = Control +ve, II = 0.5%w/w, III = 1%w/w, IV = 1.5%w/w, V = 2%w/w. **Desc: Score 1:** dentinal bridge is completely formed; **Score 2:** dentinal bridge covering more than half of the exposed area; **Score 3:** dentinal bridge initiation, not covered half of the exposure area; **Score 4:** no dentinal bridge. NS: non-significant.

* *P* ≤ 0.05 mean significant variation exists.

- **7 days:** No reparative dentine bridge formation was observed in any group; all samples scored 4 (no formation), with no significant differences between groups (*P* = 1.000).
- **14 days:** Group with 1%w/w showed initial partial dentine bridge formation in all samples; group IV showed partial formation in half of samples, while control positive, 0.5%w/w, and 2%w/w groups showed none. Significant differences existed (*P* = 0.000), with 1%w/w differing significantly from others.
- **30 days:** Complete dentine bridge formation was observed in all samples with 1%w/w and most of 1.5%w/w (83.3%), while control positive and 0.5%w/w showed partial formation in half of the samples. Group with 2%w/w showed no formation. Significant differences were noted (*P* = 0.000); group with 1%w/w differed significantly from others except 1.5%w/w.
- **60 days:** Complete dentine bridges were found in all samples of 1%w/w, most of 1.5%w/w (83.3%), and majority of control positive (50%) and 0.5%w/w (66.7%). Group with 2%w/w remained significantly different with no bridge formation (*P* = 0.000).

2. Comparison between the effect of each capping group on the reparative dentine barrier score at different evaluation periods

Friedman’s test revealed statistically significant improvements over time in reparative dentine bridge continuity for groups control positive, 0.5%w/w, 1%w/w, and 1.5%w/w (*P* = 0.001, 0.001, 0.000, and 0.001, respectively), while group V showed no significant change (*P* = 0.392) (Table 3). Pairwise comparisons indicated that reparative dentine formation began by day 30 in half of the samples in control positive and 0.5%w/w, progressing to complete formation by day 60 in more than half the samples. Group with 1%w/w demonstrated earlier induction at 14 days with partial dentine bridge formation, which was completed by day 30 in all samples. Group with 1.5%w/w showed initial formation at 14 days in half the samples, with most samples showing completion by day 30. Group with 2%w/w exhibited minimal reparative dentine formation, with only 16.7% of samples showing initiation by day 60 (Table 3).

Regarding inflammatory tissue response and hard tissue formation at 7 days, there was a significant difference in inflammation but no difference in dentine bridge formation between control and experimental groups (*P* = 0.000 and *P* = 1.000, respectively) (Tables 1 and 3). At 60 days, pairwise comparison showed significant differences in inflammatory response between control and groups with 1% and 1.5% w/w SeNPs, while reparative dentine bridge formation remained similar.

## Discussion

### Inflammatory response

The success of DPC is influenced by the presence or absence of pulp inflammation, with the ideal capping material inducing a suitable host response without causing toxicity or excessive inflammation. Although certain materials may initially intensify inflammation and induce superficial necrosis, long-term clinical success depends on the pulp’s ability to resolve this response and initiate reparative dentinogenesis [17]. MTA, although commonly used in vital pulp therapy, does not fully meet the criteria for an ideal pulp-capping material and may induce inflammatory changes and tissue necrosis [18].

Selenium nanoparticles, are identified for their antioxidant effects, play a role in bones grow and tissues heal by influencing factors like fibroblast growth factor (FGF) and vascular endothelial growth factor (VEGF), which help new blood vessel formation and controlled inflammation [19, 20]. This study, looked at how different amounts of SeNPs mixed into (MTA) affected the pulp tissue in dog teeth that had been mechanically exposed.

Dogs were chosen for these experiments because their teeth are quite similar to human dental anatomy growth, and how they heal, follows the ISO 7405:2008 standards, which help reduce the number of animals used. Male dogs between 12 and 18 months old to were used to make sure their roots were fully developed and to minimize hormonal influence on outcomes [10, 11].

Histological evaluation remains the gold standard for assessing pulp status, as clinical and radiographic assessments may not detect chronic inflammation. Thus, this study employed histopathological analysis to evaluate inflammatory response, necrosis, and reparative dentine formation, critical parameters for determining the biological efficacy of MTA combined with SeNPs over time.

This study observed the pulpal healing response when treated with MTA mixed with SeNPs, inspection at 7, 14, 30, and 60 days. These time points show early tissue reactions, odontoblast differentiation, and when a hard tissue barrier forms [21]. or a pulp capping material to work well, it needs to minimize inflammation, necrosis while encouraging a calcified bridge to form [22]. Healing the pulp involves controlled inflammation, and transform undifferentiated cells into odontoblasts [23]. After pulp exposure, inflammation and necrosis are pretty common because of the trauma and the materials used [22].

After a week, greatest groups still had moderate inflammation and some necrosis. But the group treated with MTA comprising 1% SeNPs showed much less inflammation and no necrosis at all. This likely comes from the antioxidant and anti-inflammatory influences of SeNPs, which control harmful reactive oxygen species and cytokines [24]. On the other hand, the high alkalinity of MTA HP caused some necrosis and inflammation due to hydroxyl ions, which matches what earlier studies found [9].

The dogs presented diverse levels of inflammation, perhaps because their immune systems in their own unique ways. Remarkably, the group given 2% w/w SeNPs had inflammation after 7 days, which might be because acted like pro-oxidants, damaging mitochondria and causing oxidative stress [25]. By day 14, the control group still had moderate inflammation and some tissue necrosis, aligning with previous findings of prolonged MTA-induced long-lasting tissue reactions [9, 17].

The variances among studies could come down to the types of animal models used, how the pulp was exposed, and the way the experiments were set up [26]. Thus, using 1% w/w SeNPs appeared to have a positive anti-inflammatory impact. It helped calm down neutrophils and macrophages and adjusted cytokine levels, which points to its promise as a useful regulator for controlling pulpal inflammation and promoting healing [24].

After 30 days, all groups displayed less inflammation, which means the pulp was healing well. But the group with 2% SeNPs still had moderate inflammation, perhaps because too many nanoparticles can cause harmful effects [27]. The control and 0.5% SeNPs groups had mild to moderate inflammation, which might just be due to natural differences in immune responses between subjects [16]. The 1% SeNPs group did actually well– by day 7, and after two weeks it almost disappeared completely. By 30 and 60 days, there was no inflammation at all, displaying that the pulp inflammation was reversibility and likely caused by the procedure itself, not the material.

All groups, except for 2% SeNPs, demonstrated a typical early inflammatory response to foreign materials, followed by decreased inflammation over 60 days, indicating successful resolution of irritants and cellular debris [16]. Despite mild inflammation at 60 days in the 0.5% SeNPs and control groups, reparative dentine bridge formation was evident, reflecting bioactivity and odontoblast-like cell recruitment. Statistically significant inflammation reduction across time points suggests the initial acute inflammation phase is succeeded by reparative processes involving fibroblast recruitment and calcific barrier formation stimulated by MTA’s bioactivity, which induces hydroxyapatite production and alkaline phosphatase (ALP) release essential for tissue repair [28].

These findings line up with what Lee and colleagues found [6]; they showed that spherical SeNPs help boost mineralization. But when the concentration of SeNPs gets too high, it can cause oxidative stress and even cytotoxicity [26],, which probably explains why the 2% group had ongoing inflammation. This reflects a nonlinear dose–response, these nanoparticles seem to protect cells and help them heal, but at higher levels, they can actually work against the cells, causing harm and slowing down recovery. This kind of up-and-down response isn’t unique to SeNPs; it’s something observed with other nanoparticles used in healing.

Necrotic areas with moderate inflammation persisted up to 30 days in MTA-treated pulps, likely due to MTA’s high alkalinity and immune-mediated responses to injury and microbial challenge, potentially causing pulp cell damage and necrosis in severe cases [22]. Conversely, the 1% SeNPs group showed sustained absence of necrosis and diminished inflammation throughout the study, indicating preservation of pulp vitality and enhanced healing compared to other groups [15]. The anti-inflammatory properties of SeNPs are attributed to their antioxidant capacity, which downregulates pro-inflammatory mediators such as IL-1, TNF-α, and PGE2, with oxidase activity increasing with nanoparticle concentration and surface area [29].

Inflammatory responses in the 1% SeNPs group were significantly lower than in other groups at all times, except for a non-significant difference with the 1.5% group at 30 and 60 days. This supports the safety and efficacy of incorporating 1% SeNPs into MTA as a pulp capping agent, accelerating pulpal healing and reducing inflammation more effectively than MTA alone (current study).

### Reparative dentine formation

The present study highlights the essential role of a slight inflammatory response in initiating signaling pathways that promote progenitor cell migration, proliferation, differentiation, and subsequent reparative dentine formation, which protects the pulp tissue [30]. Severe inflammation, however, may damage healthy cells and impair regeneration. No reparative dentine bridges were observed at 7 days across all groups, likely due to the short dentinogenesis period, requiring longer pulp-material contact to stimulate odontoblastic differentiation and dentine formation [31]. The control group showed reparative dentine bridge formation beginning at 30 days, improving by 60 days, consistent with prior findings [21]. The 1% SeNPs group demonstrated accelerated reparative dentinogenesis, with 100% of samples showing early dentine matrix formation at 14 days and reduced inflammation compared to other groups, indicating enhanced odontoblastic differentiation [5, 28]. Lower concentrations of SeNPs (0.5%) and control groups showed slower dentine bridge formation, while 2% SeNPs failed to induce reparative dentine within 30 days, possibly due to cytotoxic effects from pro-oxidant activity at higher SeNPs concentrations [27, 32].

MTA is known to promotes dentine formation by triggering the release of TGF-β1 and boosting genes linked to odontoblasts, which helps lay down mineralized tissue [33]. Mild inflammation persisted up to 60 days in both the experimental and control groups, which actually makes some inflammation is part of how the pulp heals and odontoblast-like cells develop [34]. The favorable alkaline environment formed by 1% SeNPs supports ALP activity, which is key for critical mineralized tissue and pulp repair [35]. SeNPs have antioxidant effects that help protect cells by modulating ROS levels, encouraging those cells to turn into odontogenic types and form matrix. But too much SeNP can be destructive, acting as a pro-oxidant, so getting importance of dosage [27, 32].

Under the microscope, the spherical SeNPs about 52 nm in size at a 1% concentration displayed healthy pulp tissue with no signs of inflammation and a fully formed dentin bridge starting from day 14 [6]. This amount seemed to work best, helping the pulp heal quicker and better than both the control group and other doses. SeNPs able to manage oxidative stress, boost mineral growth, and keep pulp vitality [15]. After 60 days, groups looked similar in terms of new dentin formation, except the 2% SeNPs group, which showed less favorable outcomes. Overall, mixing MTA with 1% SeNPs directed to a better pulp response and quicker dentin formation compared to using MTA alone. This research suggests that low-dose SeNPs added to MTA provided superior pulp response to treat vital pulp, balancing inflammation, antioxidant effects, and tissue repair [6, 28].

The formation of the dentinal bridge at the pulp-pulp capping material interface remains debated, as it can indicate either healing or irritation. In this study, dentinal bridge formation was interpreted as a positive healing response, consistent with Zaen [28]. A critical characteristic of pulp capping materials is their ability to minimize pulpal inflammation, which is essential for biocompatibility and clinical success [18]. Significant differences were observed in tertiary dentine production and inflammatory responses between MTA alone and MTA combined with SeNPs, likely due to compositional changes and interactions between SeNPs and MTA [36]. Notably, 1% w/w SeNPs accelerated reparative dentine formation, potentially through enhanced angiogenesis and mineralization mediated by VEGF upregulation and increased pulpal blood flow, which promote osteo/odontoprogenitor cell recruitment and differentiation [19, 20].

At a 2% concentration of SeNPs, hard tissue deposited in the fibrous connective tissue far from the exposure site. This might be due to fibroblasts become activated and release extracellular ATP [37]. This fits well with what Lee et al. (2021) [6], they detected more mineralization with spherical SeNPs. On the other hand, when SeNP levels are higher, they can cause oxidative stress and cytotoxicity [27], which possibly explains persistent inflammation in 2% group. Hydrated MTA, releases Ca (OH)₂ and calcium silicate hydrate. The Ca²+ ions then react to form hydroxyapatite. Adding SeNPs leads to the formation of calcium selenite [36], which adsorbs to the hydroxyapatite surfaces and forms selenite-hydroxyapatite (SeO₃-HA). This compound not only kell bacteria but as well boosts bone metabolism and acts as a substrate for selenoproteins, which have antioxidant properties. Selenite also suppresses inflammatory cells, adding to its antibacterial benefits [38]. Nonetheless if selenium levels get too high – 3% or more– it can become toxic, damaging cells and impaired bone protein synthesis [39].

Selenite stimulates osteoblast differentiation by increasing osteocalcin, ALP activity, and collagen synthesis, counteracting oxidative inhibition and activating osteoblast signaling pathways. The reduced inflammatory response observed in the 1% SeNPs group may be attributed to their antioxidant activity, which regulates ROS and downregulates pro-inflammatory cytokines, thereby promoting reparative dentinogenesis [40]. The study confirmed its hypothesis regarding inflammation and hard tissue formation only at specific SeNP concentrations compared to MTA alone. The combination of SeNPs and MTA enhanced pulp vitality and reparative dentine formation by downregulating intracellular ROS during odontoblastic differentiation of DPSCs, thereby promoting dentine-pulp complex regeneration. Additionally, SeNPs were found to reduce setting time in a concentration-dependent manner, an important factor for effective DPC agents.

These results hint that adding 1% SeNPs to MTA could be a promising way to improve vital pulp therapy in clinical practice. The enhanced dentine repair and less inflammation, which could help retain the pulp vitality in humans. Still, even though the tests in dogs look good, further clinical trials are necessary to approve safety, optimal dose, and understand the long-term effects before using it widely in humans.

## Conclusions

In this study’s scope, adding 1% SeNPs to MTA really demonstrated superior biological performance. It helped calm early pulp inflammation, boosted healing, and sped up the formation of reparative dentine compared to using MTA alone or with higher SeNP levels. The 1% SeNP presented favorable biocompatibility, but when the amount went up to 2%, inflammation increased markedly. These results hint that a small dose of SeNPs might be a helpful boost in vital pulp therapy to keep the pulp healthy. However, further clinical investigations are required to confirm safety, optimal dosing, and long-term outcomes in human applications.

## Data Availability

The datasets used to base the conclusions of the work presented here are available from the author to whom correspondence should be addressed on request.
